# SYN‐A, a naturally derived synergist, restores pyrethroid efficacy against cabbage stem flea beetle but negatively impacts its parasitoid *Microctonus brassicae*

**DOI:** 10.1002/ps.70496

**Published:** 2026-01-06

**Authors:** Patricia A. Ortega‐Ramos, Graham D. Moores, Samantha M. Cook

**Affiliations:** ^1^ Rothamsted Research Harpenden UK; ^2^ ApresLabs Ltd Harpenden UK

**Keywords:** *Brassica napus*, insecticide, IPM, natural products, *Psylliodes chrysocephala*, rapeseed, resistance

## Abstract

**BACKGROUND:**

The cabbage stem flea beetle (CSFB, *Psylliodes chrysocephala*) has become the most significant pest of winter oilseed rape (OSR) in Europe following the 2013 ban on neonicotinoid insecticidal seed treatments. Widespread pyrethroid insecticide resistance in this pest has severely limited the primary means of control for many growers, contributing to dramatic yield losses and a decline in OSR cultivation across Europe. This study evaluated SYN‐A, a novel natural synergist derived from olive oil unsaturated fatty acids, for its potential to restore pyrethroid efficacy against CSFB while assessing impacts on the parasitoid *Microctonus brassicae*, a key natural enemy of the adult life stage.

**RESULTS:**

*In vitro* enzyme assays demonstrated that SYN‐A effectively inhibited cytochrome P450 and esterase activity – key metabolic pathways associated with pyrethroid resistance – in both CSFB and *M. brassicae* in a dose‐dependent manner. Glass vial bioassays revealed that SYN‐A significantly enhanced efficacy of the pyrethroid insecticide lambda‐cyhalothrin against CSFB, increasing mortality more than threefold compared with the insecticide alone. The synergistic effect was sufficiently strong that lambda‐cyhalothrin at 20% field rate combined with SYN‐A achieved 2.2 times greater control than full‐rate lambda‐cyhalothrin alone. Semi‐field experiments confirmed laboratory findings, with SYN‐A + lambda‐cyhalothrin treatments increasing CSFB mortality from 20% to 75% and reducing plant damage by at least 50% compared with lambda‐cyhalothrin applications. However, SYN‐A also synergized lambda‐cyhalothrin against *M. brassicae* with 100% mortality when combined with lambda‐cyhalothrin at both 20% and 100% field rates. Sex‐specific responses were evident, with female parasitoids showing greater tolerance than males to both SYN‐A and lambda‐cyhalothrin.

**CONCLUSION:**

The synergist SYN‐A can effectively restore pyrethroid efficacy against metabolic resistant CSFB populations, potentially allowing up to 80% reduction in insecticide application rates while maintaining superior control. However, the severe impacts on beneficial parasitoids highlight the need for careful implementation strategies, including precise timing of applications outside peak parasitoid activity periods and continued evaluation of non‐target effects. © 2026 The Author(s). *Pest Management Science* published by John Wiley & Sons Ltd on behalf of Society of Chemical Industry.

## Graphical abstract

SYN‐A, a naturally derived synergist, inhibited key metabolic pathways associated with pyrethroid insecticide resistance in cabbage stem flea beetle (CSFB) and its parasitoid *Microctonus brassicae*. SYN‐A restored pyrethroid efficacy against resistant CSFB allowing up to 80% reduction in application rates. Parasitoids showed reduced sensitivity to pyrethroids alone, but addition of SYN‐A had negative impacts highlighting the need for careful implementation strategies.

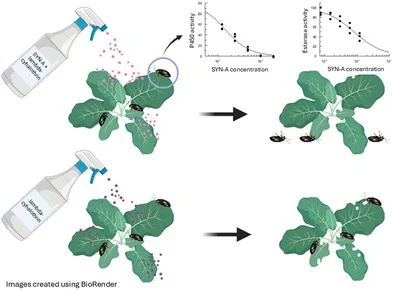

## 1 INTRODUCTION

The cabbage stem flea beetle, *Psylliodes chrysocephala* L. (CSFB, Coleoptera: Chrysomelidae) has been ranked as the most important pest of winter oilseed rape (OSR, *Brassica napus* L.) in Europe.^1^ Adult CSFB migrate into the crop in early autumn when the OSR crop is sown. They feed on the cotyledons and young leaves giving rise to ‘shot‐holing’ symptoms.^2^ Damage to the hypocotyl at the cotyledon stage or severe and sustained feeding damage to the first leaves can threaten crop establishment.^3^ Soon after arriving in the crop, adults start laying eggs in the soil, and larvae bore into the plant petioles and stem to feed throughout the winter and into late spring.^4^ When the CSFB larvae are fully developed, they exit the plant and pupate in the soil.^5^ The larvae cause severe damage to the plant causing reduced plant vigour and increased risk of frost damage and disease, stem splitting, delayed or reduced flowering, reduced yield and even plant death.^4^, ^5^, ^6^, ^7^ For a detailed description of the CSFB life cycle see Ortega‐Ramos *et al*.^8^

In the UK and Northern Europe, increasing pressure from CSFB has contributed to yield losses and complete failure of the crop in some areas.^1^, ^9^ In the south‐eastern regions of England, entire crops have been lost because of CSFB infestations, forcing farmers to either resow or abandon OSR in favour of alternative crops.^10^, ^11^ As a result, the area of OSR cultivation has declined in the past decade across Europe.^12^, ^13^ In the UK, the 2023 area of OSR was the third lowest area since 1990,^14^ resulting in a rise in OSR imports. As a consequence, the UK has moved from being an important exporter of OSR (fifth biggest exporter in the world in the 2010s) to become a net importer in the past 5 years, costing approximately £1 billion to the UK economy each year.^15^, ^16^

The decline in OSR cropping is largely due to farmers’ concerns over an inability to control CSFB populations following the ban on neonicotinoid insecticidal seed treatments.^17^ Neonicotinoid seed treatments have been used to effectively control CSFB since the early 2000s but were banned in 2013 because of concerns regarding negative effects on the environment and biodiversity.^18^, ^19^, ^20^ Following the ban on neonicotinoids, CSFB larval numbers in the UK increased tenfold, demonstrating the significant role these insecticides previously played in controlling infestations.^21^ With the ban in place, growers were left with just one class of insecticide for controlling CSFB: pyrethroids. Consequently, the mean number of insecticide applications (primarily pyrethroids) in the UK increased from 2.2 sprays in 2012, to 2.5 sprays per season in 2018;^22^, ^23^ although important, this is not as substantial as in Germany, where autumn insecticide use on OSR quadrupled between 2012 and 2019.^12^

The prolonged and extensive use of pyrethroids against CSFB has resulted in high selection pressure for insecticide resistance, leading to the development of pyrethroid resistance across Europe. The first reports of CSFB resistance to pyrethroids came from Germany in 2008.^24^, ^25^ Since then, the resistance levels have been increasing and reports of resistance spread across Europe: UK,^26^, ^27^ Denmark,^28^ France^29^ and Czech Republic.^30^ Willis *et al*.^31^ found that mean resistance in the UK increased by 23% from 2018 to 2019. This and later annual resistance monitoring have found that pyrethroid resistance was widespread across England and that there are no fully susceptible populations in the entire UK.^32^ Three different resistance mechanisms in CSFB have been described: L1014F kdr mutation in the voltage‐gate sodium channel conferring target‐site (knockdown) resistance;^25^ super‐knockdown resistance (skdr), due to the L925I/M918L mutation;^29^, ^31^ and metabolic‐based resistance.^26^, ^27^ Despite the well‐studied issue of insecticide resistance and the widely available annual resistance surveys and reports (e.g. UK‐AHDB monitoring and managing insecticide resistance project), farmers have had little choice but to continue to rely on pyrethroid insecticides for OSR cultivation. In 2022, 63.5% of OSR crops – equating to 332 332 ha – were treated with insecticides and nematicides, highlighting the ongoing dependence on chemical control methods.^33^

Given this situation, there is a growing need to develop more effective pest management options for farmers to be able to control CSFB, and other insect pests, in a sustainable and efficient way. However, there are few options in the research and development pipeline that are close to commercialization.^8^ Furthermore, given the difficulty of registering new products, ways are needed to overcome resistance and improve the efficacy of existing options. One such option involves the use of synergists, compounds that enhance the efficacy of insecticides without being toxic by themselves.^34^ Synergists act primarily by inhibiting the metabolic pathway responsible for insecticide detoxification in resistant insects, temporarily restoring a level of susceptibility.^35^, ^36^ Since the first report on enhanced insecticidal activity of pyrethrum following the addition of the natural synergist ‘sesamin’, synthetic chemical synergists such as piperonyl butoxide (PBO), diethyl maleate, verapamil, *S*,*S*,*S*‐tributyl phosphorotrithioate and methylenedioxyphenyl have been widely used for the diagnosis of insecticide resistance mechanisms;^37^ if insecticide susceptibility is restored after addition of a synergist, metabolic resistance is implied. SYN‐A is a novel, natural synergist derived from the unsaturated fatty acids present in olive oil.^38^ Like PBO, it functions by inhibiting both cytochrome P450 monooxygenases and esterase enzymes – key metabolic pathways associated with insecticide resistance in many pest species, as demonstrated in the supporting patent.^38^

A critical aspect of integrating any pesticide or synergist into a pest management strategy is the evaluation of its effects on non‐target organisms, particularly beneficial arthropods. These organisms provide essential ecosystem services, including biological control, and their conservation is key for sustainable agriculture. Synergists that enhance insecticide efficacy may also inadvertently increase toxicity to beneficial species. Therefore, rigorous studies assessing both the efficacy and ecological safety of synergists like SYN‐A – alone and in combination with insecticides – are essential before they can be recommended for widespread use in crop protection.

This study aimed to investigate the potential of a novel natural compound (SYN‐A) to synergize the pyrethroid lambda‐cyhalothrin against adult CSFB and the impact on its main parasitoid, *Microctonus brassicae*. *M. brassicae* lays its eggs inside the adult stage of CSFB,^39^ and the beetle is killed when the parasitoid larva is ready to pupate and exits the host's body.^8^ We evaluated the inhibitory effects of SYN‐A on metabolic resistance enzymes (cytochrome P450s and esterases) in both CSFB and *M. brassicae* using *in vitro* enzyme assays. We also tested the *in vivo* efficacy of SYN‐A using glass vial bioassays and semi‐field simulation. Furthermore, we characterized the dose–response relationship of pyrethrum against CSFB and examined its synergistic interaction with SYN‐A. Because it is reported that PBO, a methylenedioxyphenyl compound, synergizes both lambda‐cyhalothrin and pyrethrum^40^, ^41^ we have used it to benchmark the effects of SYN‐A.

## 2 MATERIAL AND METHODS

### 2.1 Chemicals and insecticides

SYN‐A is a mixture of unsaturated fatty acids as described in patent WO2017/005728. Stock solutions of SYN‐A (1%) were prepared in acetone. Formulated pyrethroid insecticide – lambda‐cyhalothrin, Hallmark Zeon (100 g L^−1^ lambda‐cyhalothrin) – was purchased from Merck (Sygma‐Aldrich Co, Guillingham, UK); ‘Breaker Natur’ (formulated pyrethrum) was purchased from Certis‐Belchim (Saronno, Italy). All other chemicals including technical grade lambda‐cyhalothrin (PESTANAL, analytical standard) and PBO (PESTANAL, analytical standard) were purchased from Merck (Sigma‐Aldrich Co, Guillingham, UK).

### 2.2 Insects

CSFB adults were collected from freshly harvested OSR grain stores at Rothamsted Research experimental farm and from nearby commercial farms using hand‐held, battery‐powered aspirators. *M. brassicae* parasitoids were initially reared from field‐collected beetles and later reared under laboratory conditions (adapted from Beran *et al*.^42^). Continuous cultures of both beetles and parasitoids were maintained together within mesh cages (35 × 17.5 × 17.5 cm) in controlled environment cabinets (22 °C, 80% relative humidity, 12:12 h light/dark photoperiod). Fresh OSR leaves (*Brassica napus* cv. Apex) were provided as a food source twice a week for adult beetles and parasitoids were provided with a 50:50 honey–water solution.

### 2.3 Enzyme inhibition assays

#### 2.3.1 Insect homogenization

Ten adult CSFB or *M. brassicae* parasitoids were homogenized in 500 μL of 0.2 m phosphate buffer (pH 7.0) and centrifuged at 10 000 *g* for 10 min, and the supernatant was taken as the enzyme source for esterase activity. For oxidase activity, 20 adult beetles or parasitoids were homogenized on ice in 250 μL homogenization buffer (0.1 m phosphate buffer, pH 7.6, containing 1 mm EDTA, 1 mm dithiothreitol, 1 mm phenylthiourea, 1 mM phenylmethylsulfonyl fluoride) and then diluted with the same buffer to give a final volume of 1 mL. This was centrifuged at 10 000 *g* for 10 min, and the supernatant was taken as the enzyme source.

#### 2.3.2 Esterase inhibition assay

Aliquots of CSFB or *M. brassicae* parasitoid supernatant were incubated for 10 min with serial dilutions of SYN‐A to give final concentrations of between 0.15% and 0.001%, and total esterase activity remaining was measured in 96‐well microplates using a colorimetric assay modified from Pocker and Stone.^43^ Briefly, the rate of hydrolysis of 4‐nitrophenyl octanoate was assayed at 405 nm. Kinetic assays were performed using a Thermomax microplate reader (Molecular Devices, Menlo Park, CA, USA) reading for 5 min at 10‐s intervals. The integrated ‘Softmax’ software was used to fit a linear regression to the kinetic plots. Half‐maximal inhibitory concentration (IC_50_) values were calculated using Grafit 3.0 (Leatherbarrow, Erithicas software). Enzyme in the presence of acetone was used as the uninhibited control. All assays were repeated in triplicate.

#### 2.3.3 P450 inhibition assay

Aliquots of beetle or parasitoid supernatant were incubated for 10 min with serial dilutions of SYN‐A to give final concentrations of between 0.02% and 0.00125%. *O*‐Deethylation of 7‐ethoxycoumarin was measured according to Ullrich and Weber^44^ and adapted to the microplate format as described by de Sousa *et al*.^45^ Kinetic assays were performed using a SpectraMax Gemini EM fluorescence microplate reader (Molecular Devices) reading for 60 min at 30‐s intervals using an excitation wavelength of 370 nm and an emission wavelength of 460 nm. The integrated ‘Softmax’ software was used to fit a linear regression to the kinetic plots. IC_50_ values were calculated using Grafit 3.0 (Leatherbarrow, Erithicas software).

### 2.4 Glass vial bioassays

To test the effects of the different chemical treatments on CSFB and *M. brassicae*, glass vial bioassays based on Insecticide Resistance Action Committee Method 31 (www.irac-online.org/methods/weevils-and-flee-beetles/2014) were used. Glass vials (14 mL; 7 cm tall and 2 cm in diameter) (S Murray and Co, UK) were prepared by coating the inner surface with different concentrations and mixtures of the insecticide (lambda‐cyhalothrin or pyrethrum) and/or the synergist (SYN‐A or PBO) treatments and allowed to fully dry before the addition of insects. Stock solutions were prepared by diluting the technical grade synergists and insecticides in technical grade acetone. Vials coated with acetone alone were used as controls. Individuals of the test insects (ten CSFB or one *M. brassicae*) were transferred to the treated vials and these were closed with a plastic lid and left at 20 °C under a 12:12 h light/dark photoperiod. After 24 h, both CSFB and parasitoids were scored and then transferred to untreated glass vials without a lid under upturned 200 mL plastic disposable cups (VWR International Ltd, Dublin, Ireland), to allow them to recover. After a further 24 h the insects were scored again. Three scoring categories were used: ‘mobile’ (capable of jumping or walking in a coordinated way in case of CSFB or walking/flying for parasitoids); ‘affected’ (incapable of jumping or coordinated movement); or ‘dead’ (no movement).

To evaluate the effects of different pesticide and synergist combinations on adult CSFB, ten beetles were placed in each vial, with each treatment replicated three times. For parasitoids, because of the low numbers available for testing, only one individual was used per vial. Six parasitoids of each sex per treatment were tested to assess the SYN‐A and PBO dose response (*n* = 12) but only three female parasitoids were used to assess the effect of synergists in combination with insecticides. Doses equivalent to 10%, 15%, 50% and 100% of the recommended field rate (equivalent to 7.5, 11.3, 37.5 and 75 ng cm^−2^) were used to test SYN‐A and PBO dose response on *M. brassicae*. To assess the dose–response of pyrethrum on CSFB, doses corresponding to 15, 75, 150 and 375 ng cm^−2^ were used. Each assessment included replicated control vials that were treated with acetone only.

The synergists (SYN‐A and PBO) were tested in combination with the insecticide. Preliminary assays showed no significant difference between pre‐exposure and co‐application (mixing); therefore, the mixing method was adopted for all experiments, because it more closely reflects typical field practice where farmers apply synergists and insecticides together in a single spray application. When assessing the combined effects of synergists and insecticides, two insecticide doses equivalent to 20% and 100% of the recommended field application rate of lambda‐cyhalothrin (7.5 g a.i. ha^−1^) were used. The synergist dose used against CSFB was 11 μg cm^−2^, as PBO had been shown to not confer control mortality at this concentration.^26^ For *M. brassicae*, the synergist dose used was 1.1 μg cm^−2^, because it represented the maximum dose that did not cause mortality, as determined from the dose–response assay.

### 2.5 Field simulation

A field simulator trial was performed in seminatural conditions to assess the effects of pyrethrum and pyrethroid (lambda‐cyhalothrin) insecticides, with and without the synergist SYN‐A, on CSFB. Groups of six OSR cv Apex plants at the two‐true leaf growth stage (BBCH 12),^46^ were grown in 17.5 × 33 × 6 cm seed trays and topically treated using a track sprayer (nozzle, 110015VK; height, 50 cm; pressure, 179 kPa) to simulate tractor‐mounted spraying. The experimental treatments included: (i) lambda‐cyhalothrin + SYN‐A, (ii) pyrethrum + SYN‐A, (iii) lambda‐cyhalothrin alone, (iv) pyrethrum alone, (v) SYN‐A alone, and (vi) a water control treatment. Lambda‐cyhalothrin was applied at 0.075 L ha^−1^ (the recommended field rate), pyrethrum at 1.1 L ha^−1^, and SYN‐A at 1.6 L ha^−1^. Tween 20 was added to all treatments as an adjuvant at 0.5% (v/v). Each treatment was diluted in distilled water to the appropriate spray volume, whereas control plants received distilled water only.

Following treatment, OSR plants were allowed to air‐dry for 2 h before CSFB exposure. The sexes of the beetles were determined prior to the experiment^47^ and individually exposed to their respective treatments for 48 h. A total of 120 beetles (60 males and 60 females) were tested, with each treatment replicated 20 times: 10 replicates for females and 10 for males. After introduction of a beetle into the tray, the tray was covered with a clear ventilated plastic lid to prevent the beetles from escaping. Trays were randomly allocated to positions within benches in a controlled environment room (20 °C, 12 h light) according to a ten‐block (5 benches × 2 days) randomized complete block design. After the exposure period, the individual beetles were recovered from the trays and scored as alive (unaffected), affected (immobilized but not dead), or dead as described above. Missing beetles were recorded as such. Feeding damage on the plants in the tray was assessed by counting the number of plants per tray with feeding symptoms and recording the mean number of feeding holes per leaf.

### 2.6 Statistical analyses

All statistical analyses were conducted in R Studio 2024.12.0.^48^ Data visualization was performed using the ‘ggplot2’ package. Dose–response analysis of the test chemicals and the concentration required to kill 50% of the population (LD₅₀) estimation were carried out using the ‘drm’ and ‘ED.drc’ functions from the ‘drc’ package.^49^ For LD₅₀ estimation and subsequent analyses, individuals that were scored as ‘affected’ were included with those that were ‘dead’, assuming that under field conditions, their likelihood of survival would be negligible because of reduced mobility and increased predation risk.

A Generalized Linear Model (GLM) with negative binomial distribution was used to assess the effect of different treatments (synergists combined with varying concentrations of lambda‐cyhalothrin or pyrethrum) on CSFB mortality rates in glass vial assays using the ‘glm.nb’ function from the ‘MASS’ package. Pairwise comparisons were conducted using the ‘emmeans’ package. A GLM with binomial distribution (probit link function) was used to analyse the effects of treatments on *M. brassicae* mortality using the ‘glm’ function and the ‘anova; function was used to generate the analysis of deviance table.

To assess the effect of treatment on CSFB mortality in the simulated field experiment, a GLM with a binomial distribution was fitted using the ‘glm’ function. Model significance was evaluated using an analysis of deviance table generated with the ‘anova’ function (chi‐squared test). Post‐hoc pairwise comparisons were conducted using the ‘emmeans’ package to adjust for multiple comparisons. The effect of treatments on the proportion of CSFB‐damaged plants was assessed using a GLM with a binomial distribution. A linear regression model was used to analyse the effect of treatment on the average number of holes per leaf. The number of holes per leaf was log(*x* + 1)‐transformed to meet normality assumptions of the model.

## 3 RESULTS

### 3.1 Laboratory experiments

#### 3.1.1 Esterase and P450 inhibition

The synergist SYN‐A was found to effectively inhibit activity of key metabolic enzymes correlated to pyrethroid detoxification in both CSFB and *M. brassicae*. In CSFB, SYN‐A exhibited a dose‐dependent inhibition of cytochrome P450 (Fig. 1(A)) and esterase activity (Fig. 1(B)). Similarly, SYN‐A inhibited P450 (Fig. 1(C)) and esterase (Fig. 1(D)) activity in *M. brassicae*, also in a dose‐dependent manner. The IC₅₀ values indicate that *M. brassicae* enzymes were more sensitive to SYN‐A compared with CSFB, suggesting potential non‐target effects of the synergist on beneficial parasitoids.

**Figure 1:**
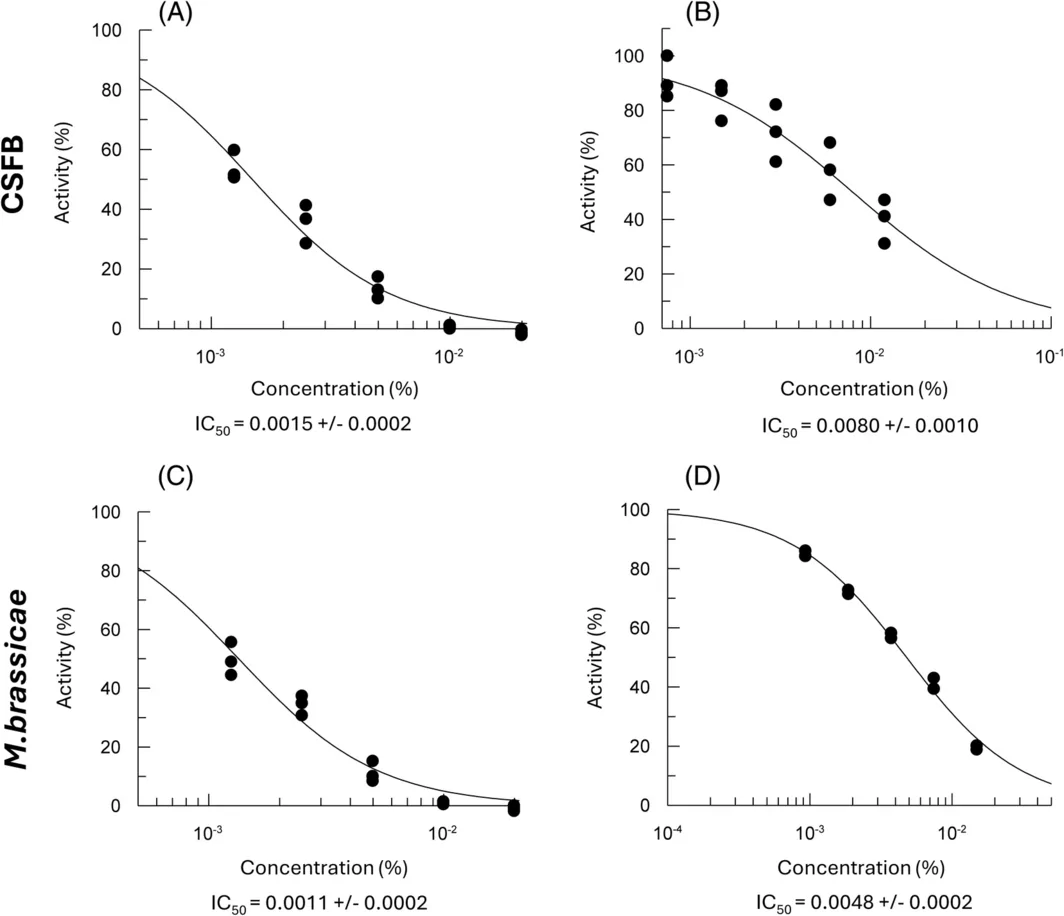
Relationship between the inhibition of cytochrome P450 (P450) (A) and esterase activity (B) in cabbage stem flea beetle (*Psylliodes chrysocephala*) pest insects and concentration of the organic synergist SYN‐A. Inhibition of P450 (C) and esterase activity (D) in the parasitic wasp *Microctonus brassicae* by SYN‐A. Enzyme activity was measured as a function of SYN‐A concentration, with IC₅₀ values indicating the concentration required to inhibit 50% of enzymatic activity.

#### 3.1.2 Synergist dose–response in *M. brassicae*

The dose–response analysis showed similar LD₅₀ values for PBO (10.68 μg cm^−2^, SE = 6.13) and SYN‐A (12.08 μg cm^−2^, SE = 2.03) in *M. brassicae*, (Fig. 2). There were marginal sex‐specific differences; males exhibited a lower LD₅₀ [PBO: 11.85 μg cm^−2^ (SE = 0.08); SYN‐A: 9.02 μg cm^−2^ (SE = 0.09)] than females, which had higher LD₅₀ to both synergists [PBO: 12.89 μg cm^−2^ (SE = 0.23); SYN‐A: 20.65 μg cm^−2^ (SE = 0.74)].

**Figure 2:**
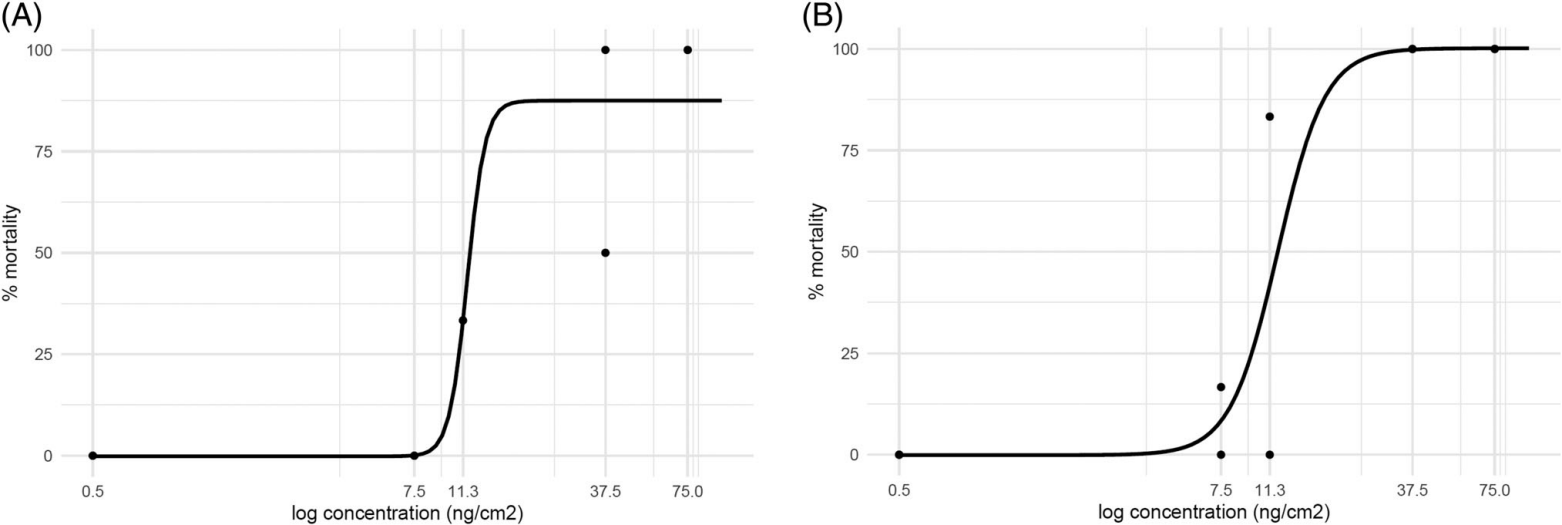
Dose–response curves showing the effect of synergists synthetic piperonyl butoxide (PBO) (A) and organic SYN‐A (B) on *Microctonus brassicae* mortality. Mortality (%) is plotted against the logarithm of the applied synergist concentration (ng cm^−2^). Black dots represent observed mortality at each concentration, and the fitted curves represent the estimated dose–response relationship.

#### 3.1.3 Effects of synergists on CSFB susceptibility to insecticides: glass vial bioassays

Exposure of CSFB to SYN‐A or PBO significantly increased pest sensitivity to lambda‐cyhalothrin both at 20% and 100% the field rate (Fig. 3). Results from the negative binomial regression model showed significant differences in the mortality rates between treatments (Likelihood Ratio Test = 264.51, df = 12, *P* < 0.001).

**Figure 3:**
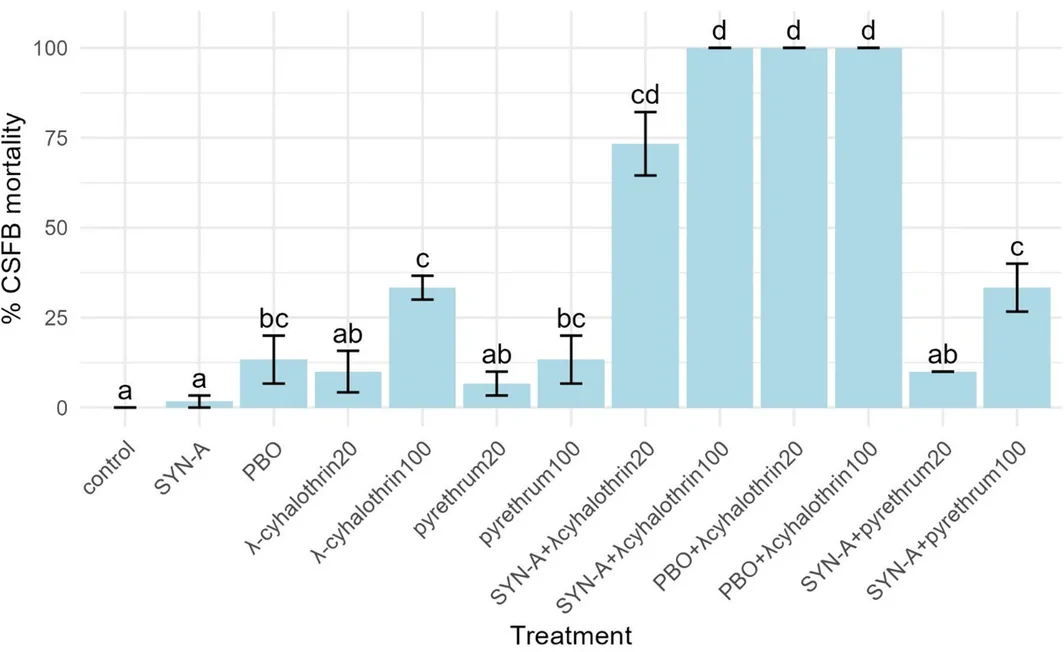
Mean (± standard error) mortality (%) of cabbage stem flea beetle (*Psylliodes chrysocephala*) following exposure to two synergists [synthetic piperonyl butoxide (PBO) and SYN‐A] and two insecticides (organic pyrethrum or the synthetic pyrethroid *λ*‐cyhalothrin) at 20% and 100% of the recommended field rate in glass vial assays. The numbers 20 and 100 after the treatment name indicate the field rate at which the insecticide was applied (20% or 100% of the recommended field rate).

Both PBO and SYN‐A significantly increased the susceptibility of CSFB to lambda‐cyhalothrin, and there were no significant differences between using PBO or SYN‐A together with lambda‐cyhalothrin at 100% field rate (*z*‐ratio = 0, *P* = 1). The CSFB mortality increased significantly when using SYN‐A with lambda‐cyhalothrin compared with the use of lambda‐cyhalothrin on its own, both at 20% and 100% of the field rate (*z*‐ratio = 3.78, *P* = 0.01 and *z*‐ratio = 2.28, *P* < 0.5, respectively). When used in combination with SYN‐A, lambda‐cyhalothrin resulted in more than three times higher mortality than lambda‐cyhalothrin on its own (Fig. 3). Furthermore, the analysis showed that using SYN‐A with lambda‐cyhalothrin at 20% field rate resulted in 2.2 times higher mortality than full field rate of lambda‐cyhalothrin on its own. Although there were no significant differences between SYN‐A + lambda‐cyhalothrin at 100% and SYN‐A + lambda‐cyhalothrin at 20% (*z*‐ratio = 0.62, *P* = 1), SYN‐A + lambda‐cyhalothrin at 100% conferred 1.37 times higher mortality than SYN‐A + lambda‐cyhalothrin at 20%.

There was no significant difference in CSFB mortality when exposed to pyrethrum at 100% field rate and the control (*z*‐ratio = −0.01, *P* = 1). Although SYN‐A doubled the efficacy of pyrethrum at 100% field rate against CSFB (Fig. 3), the control level achieved was only around 30% mortality, although this was equal to that reached by the field rate of lambda‐cyhalothrin alone.

No statistical differences were found between the control and the synergists on their own (PBO, *z*‐ratio = −0.07, *P* = 1; SYN‐A *z*‐ratio = −0.06, *P* = 1); exposure to synergist alone (without insecticide) did not show any significant mortality on CSFB.

#### 3.1.4 Effects of synergists on *M. brassicae*: glass vial bioassays

In glass vial bioassays, synergists increased the sensitivity of *M. brassicae* to lambda‐cyhalothrin, although results should be interpreted with caution given the low sample size (*n* = 3 per treatment). The binomial model revealed significant differences in mortality between treatments (*χ* ^2^ = 28.86, df = 8, *P* < 0.001). Synergists alone caused no significant increase in mortality compared with controls, but when combined with lambda‐cyhalothrin at 20% field rate, both SYN‐A and PBO resulted in 100% parasitoid mortality (Fig. 4). By contrast, *M. brassicae* showed tolerance to lambda‐cyhalothrin alone, with 100% survival at 20% field rate and ~ 33% survival at 100% field rate.

**Figure 4:**
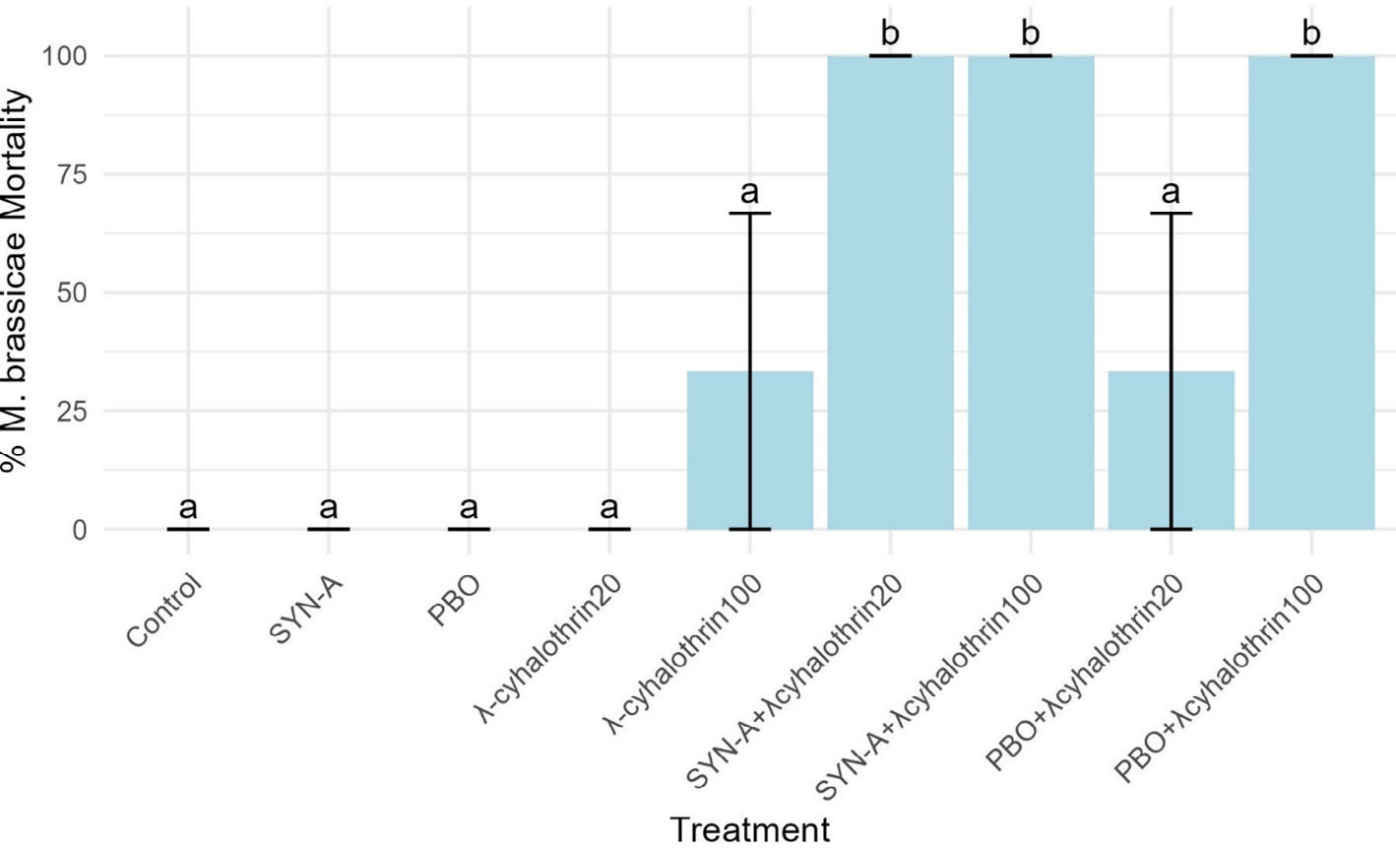
Mean (± standard error) mortality (%) of *Microctonus brassicae* adults following exposure to the synthetic pyrethroid insecticide *λ*‐cyhalothrin alone or in combination with synergists [piperonyl butoxide (PBO) and SYN‐A] at 20% and 100% of the recommended field rate in glass vial assays. The numbers 20 and 100 after the treatment names indicate the percentage of the field rate at which *λ*‐cyhalothrin was applied.

### 3.2 Simulated field experiment

Significant differences in CSFB mortality between treatments (*χ* ^2^ = 45.65, df = 5, *P* < 0.001) were observed for beetles in the simulated field experiment. Pyrethrum alone or in combination with SYN‐A conferred only approximately 5% mortality; there was no difference in CSFB mortality when exposed to SYN‐A + pyrethrum or pyrethrum on its own (*z*‐ratio = −0.09, *P* = 1). By contrast, SYN‐A significantly enhanced the efficacy of lambda‐cyhalothrin, increasing mortality from 20% to 75% (Table 1). Post‐hoc comparisons indicated that the combination of SYN‐A + lambda‐cyhalothrin resulted in significantly higher mortality than lambda‐cyhalothrin alone (*z* = −2.53, *P* < 0.1).

**Table 1:** Percentage mortality of cabbage stem flea beetle (*Psylliodes chrysocephala*) in a simulated field experiment after 48 h of exposure to oilseed rape plants treated with two different synergists (SYN‐A and piperonyl butoxide) and two insecticidal treatments (pyrethrum and synthetic pyrethroid, *λ*‐cyhalothrin), showing the number of beetles tested, beetles recovered, survival status, and calculated mortality of those found

Treatment	No. beetles tested	Total no. beetles found	Found alive	Found dead	Mortality of those found (%)
Control	20	14	14	0	0
*λ*‐Cyhalothrin	20	10	8	2	20.00
Pyrethrum	20	18	17	1	5.56
SYN‐A	20	16	16	0	0
SYN‐A + *λ*‐Cyhalothrin	20	16	4	12	75.00
SYN‐A + Pyrethrum	20	16	15	1	6.25

The proportion of CSFB‐damaged plants differed significantly among treatments (*χ* ^2^ = 34.55, df = 5, *P* < 0.001) (Fig. 5(A)). Plants treated with SYN‐A + lambda‐cyhalothrin (*z*‐ratio = −3.24, *P* < 0.001) had significantly lower proportions of damage compared with other treatments. The number of feeding holes per leaf also varied significantly across treatments (*F*_(5,114)_ = 4.112, *P* < 0.005; Fig. 5(B)). Plants treated with lambda‐cyhalothrin alone (*t* = −3.203, *P* < 0.05) and SYN‐A + lambda‐cyhalothrin (*t* = −4.492, *P* < 0.001) had significantly fewer feeding holes than the control. However, because of the low beetle recovery rate in the lambda‐cyhalothrin treatment only, the lower number of damaged plants might be due to absence of beetles (escapees). The combination of SYN‐A + lambda‐cyhalothrin enhanced the efficacy of lambda‐cyhalothrin, reducing both the proportion of damaged plants and the number of feeding holes by at least 50% compared with lambda‐cyhalothrin alone.

**Figure 5:**
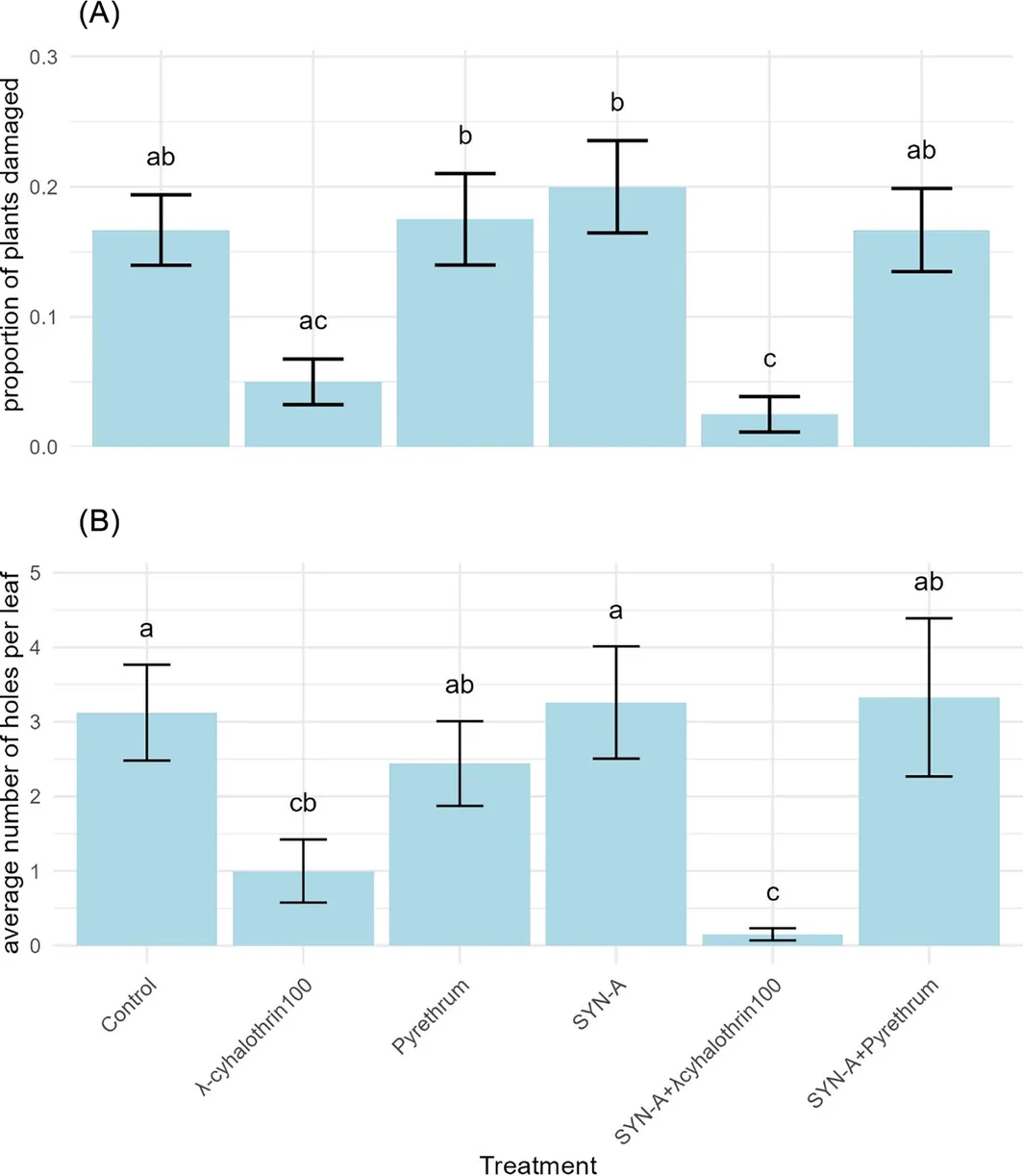
Oilseed rape plant damage caused by cabbage stem flea beetle (*Psylliodes chrysocephala*) exposed to plants treated with two synergists [SYN‐A and piperonyl butoxide (PBO)] and two insecticidal treatments [organic pyrethrum and synthetic pyrethroid (*λ*‐cyhalothrin)]. (A) Proportion of plants with damage symptoms. (B) Average number of feeding holes per leaf (considering both damaged and undamaged leaves). Error bars represent the standard error.

## 4 DISCUSSION

The synergist SYN‐A (an extract containing unsaturated fatty acids from olives) was found to effectively inhibit cytochrome P450 and esterase activity of CSFB, key enzymes responsible for pyrethroid insecticide metabolism. Exposure of CSFB to SYN‐A significantly increased sensitivity to the synthetic pyrethroid (lambda‐cyhalothrin) both at 20% and 100% the field rate. When used in combination with SYN‐A, lambda‐cyhalothrin caused more than three times higher mortality than on its own against CSFB in laboratory tests, and simulated field trials showed a corresponding 3.75‐fold boost in control. Furthermore, plant damage was reduced by at least 50% when applying lambda‐cyhalothrin + SYN‐A relative to lambda‐cyhalothrin alone. These results show that by using SYN‐A as a synergist, not only are resistance mechanisms overcome, allowing a resistant pest that previously survived sprays to be killed, but also mortality is achieved with lower doses of insecticide than current field rates. In practice, this suggests that SYN‐A could allow control of CSFB with far lower insecticide inputs and could help to achieve ambitious European Union (EU) targets to reduce insecticide use^50^ without compromising control. With few new actives coming to market, and others being lost to changes in registration, compounds like SYN‐A may extend the useful life of existing pyrethroids and other chemistries by overcoming resistance mechanisms.

Although we have shown in the *in vitro* enzyme assays that SYN‐A inhibits cytochrome P450 and esterase activity of CSFB and *M. brassicae*, it has not been shown conclusively whether the observed synergism was due to the inhibition of these metabolic enzymes alone, or whether other factors, such as increased uptake of insecticide through the insect cuticle, added to the observed increase of mortality. The mode of action of fatty acids when topically applied to soft‐bodied insects is generally perceived to be via disruption of the cuticle^51^ followed by rapid desiccation.^52^ However, in the glass vial assays both the PBO and SYN‐A were added in acetone, which was then allowed to fully dry before the addition of insects, minimizing physical effects on the cuticle. Further investigations to characterize more fully the mechanisms by which SYN‐A exerts its effects will be conducted in future experiments.

Pyrethrum was included in our experiments as a potential organic option to pair with SYN‐A, which is also naturally derived, and together would have offered a more sustainable alternative to synthetic pyrethroids. However, our results indicate that pyrethrum, either alone or in combination with SYN‐A, is not a reliable option to control CSFB under the tested conditions. When used with pyrethrum in the glass vial assays, SYN‐A showed clear synergism, increasing sensitivity to pyrethrum insecticide; however, this effect was less evident in the simulated field experiment. Several factors could explain this discrepancy. First, pyrethrum is unstable under ultra‐violet light^53^ being rapidly decomposed to non‐toxic metabolites; without the protection of the glass vials it is probable that the pyrethrum was broken down more rapidly under the lighting regime in the simulated field trial than in the glass vial assays, and this resulted in all the pyrethrum treatments (pyrethrum alone, pyrethrum + SYN‐A) conferring very little mortality. It must also be acknowledged that the glass vial and simulated field experiments were performed at different times, 1 year apart, and although beetles were collected from the same region, population‐level differences in susceptibility cannot be excluded. Although the observed effects of SYN‐A were consistent and informative, the limited sample size (*n* = 20) means these results should be interpreted with caution. Larger‐scale trials under real field conditions will be essential to confirm the robustness of these results.

Our bioassays also revealed serious non‐target risks. Our results show that SYN‐A has similar effects on *M. brassicae*, the parasitoid of adult stage CSFB, compared with its CSFB host – inhibiting cytochrome P450 and esterase activity and affecting the parasitic wasps even at low doses. Although the observed trend towards complete mortality when synergists were combined with lambda‐cyhalothrin was consistent across all replicates, these results should be interpreted with caution given the low statistical power (*n* = 3 per treatment for synergist–insecticide combinations). Furthermore, in the glass via bioassays, parasitoids were tested individually, whereas CSFB were exposed in groups of ten per vial. A single wasp in a confined space is therefore likely to encounter a greater proportion of the treated surface and therefore receive a higher effective dose of active substance than grouped insects. This difference in exposure density could have contributed to the higher apparent sensitivity of *M. brassicae* than CSFB. Nonetheless, the clear dose–response relationships observed for both synergists and the consistency with the enzyme inhibition data suggest these findings should be taken into consideration in risk assessments. Future studies with larger sample sizes would be valuable to confirm these observations and better characterize the extent of non‐target impacts.

We observed that female wasps were consistently more tolerant of SYN‐A and lambda‐cyhalothrin than males, indicating sex‐specific differences in susceptibility. Females of *M. brassicae* survived SYN‐A doses that killed most males, but even the higher female tolerance did not prevent complete mortality of the population under combined SYN‐A + lambda‐cyhalothrin exposure. This observation aligns with findings by Rathman *et al*.,^54^ who reported higher susceptibility in male *Diglyphus begini* (Hymenoptera: Eulophidae) than in females when exposed to various insecticides, including methomyl and permethrin in laboratory bioassays. Similarly, Carrière^55^ noted that in haplodiploid arthropods, males are generally twice as susceptible to pesticides than females. The haplodiploid genetic system of hymenopterans may contribute to these differences. In these systems, males are haploid and possess only a single allele for each gene, including those conferring pesticide resistance, whereas females are diploid and can carry two alleles. Carrière^55^ proposed that this genetic structure means that: (i) females could have greater detoxification enzyme production making them less susceptible; and (ii) there could also be a sexual dimorphism in gene expression, such that expression of genes coding for pesticide resistance would be generally greater in females. Size dimorphism is often also considered a factor in differences in pesticide susceptibility, with larger individuals (typically females) exhibiting greater tolerance than smaller ones.^56^ However, for the Ichneumonidae family and specifically in *M. brassicae*, males are slightly larger than females, with mean body lengths of 2.6 and 2.3 mm, respectively,^39^ suggesting that size alone does not account for the observed differences.

We also observed that *M. brassicae* individuals showed high survival when exposed to lambda‐cyhalothrin insecticide alone – essentially all survived 20% field rate, and ~75% survived the full rate. As there is no baseline ‘susceptible’ population for comparison it is not possible to assume pyrethroid resistance *per se*. However, to our knowledge this is the first report of reduced sensitivity to lambda‐cyhalothrin resistance in this species. The emergence of insecticide resistance in natural enemies is an increasingly recognized phenomenon, with a notable rise in reported cases since the 1960s.^57^ Resistance to insecticides has been observed in several field populations of parasitoid wasps, such as *Diadegma insulare* (Cresson) (Hymenoptera: Ichneumonidae),^58^ *Cotesia plutellae* Kurdjumov (Hymenoptera: Braconidae)^59^ and *Oomyzus sokolowskii* (Kurdjumov) (Hymenoptera: Eulophidae),^60^ all endoparasitoids of *Plutella xylostella*; or *Diaeretiella rapae* the dominant parasitoid of *Brevicoryne brassicae* in OSR and other brassica crops.^61^ The development of resistance in endoparasitoids like *M. brassicae*, which lay their eggs inside the host, is often influenced by the insecticide exposure of their hosts.^59^ Pesticide resistance is a positive trait in natural enemies and represents an opportunity to improve the simultaneous use of two very valuable tools in pest management: toxicant insecticides (if the pest is not resistant) and biological control.^62^ Our results highlight the importance of monitoring resistance development in beneficial arthropods and considering sex‐specific responses when evaluating the impacts of insecticides.

The incorporation of natural synergists such as SYN‐A into CSFB management offers the potential to restore pyrethroid efficacy and reduce both the dose and frequency of insecticide applications in OSR crops. For example, lambda‐cyhalothrin inputs could be decreased by up to 80% (reducing the field rate to 20%) while maintaining or even improving control relative to full‐rate treatments. However, although this may help to meet policy targets on pesticide reduction^51^ it does not necessarily translate to a proportional reduction in environmental impact. The synergist fundamentally increases the toxicity per unit of insecticide applied to both target and non‐target organisms – shifting the toxicity baseline. To truly assess the benefits and risks of synergist‐based approaches we must consider not only the quantity of insecticide applied, but also the increased potency and their effects on non‐target organisms. Furthermore, reliance on broad‐spectrum insecticides carries risks for beneficial arthropods and the ecosystem services they provide. In Europe, insecticide registration includes standardized tests on selected non‐target species: pollinators (honey bees, bumble bees, solitary bees)^63^ and one of four predators (*Orius laevigatus*, *Chrysoperla carnea*, *Coccinella septempunctata* or *Aleochara bilineata*).^64^ However, these species may not represent the key natural enemies present in the crop environment at the time of application or be an important natural agent to control the targeted pest. Therefore, understanding the responses of locally active natural enemies, like *M. brassicae* for CSFB, is essential when evaluating the broader ecological impact of incorporating synergists such as SYN‐A into pest management.

Nevertheless, SYN‐A's potent synergism offers a path forward within an integrated pest management (IPM) framework if applied judiciously. EU policy explicitly states that IPM includes the ‘use of plant protection products (…) to levels that are economically and ecologically justified and reduce or minimize risks to human health and the environment’ (Directive 2009/128/EC).^65^ IPM emphasizes an ordered decision‐making process: monitoring pest thresholds, prioritizing preventive measures and non‐synthetic chemical controls, and using pesticides only when necessary and in the most targeted way.^66^ Under these principles, combining SYN‐A with reduced rates of existing insecticides could support IPM objectives by dramatically cutting insecticide load. Indeed, Barzman *et al*.^66^ note that ‘reduced pesticide use, in terms of frequency, spot spraying, or dose reduction, is a recognized tactic along the IPM continuum that can be combined with other ones’. By restoring pyrethroid efficacy in resistant CSFB, SYN‐A might allow growers to revert to lower doses or fewer applications of pyrethroids (or even switch back from more harmful classes), thereby delaying the need for managing resistance and critically, giving time for alternatives to be developed and commercialized.^8^, ^51^ Achieving these benefits requires balancing enhanced pest mortality against conservation of beneficial species by mitigating risks to non‐targets. Temporal targeting – applying synergist–insecticide mixtures outside the peak activity windows of key parasitoids – can reduce non‐target exposure if coupled with detailed phenological monitoring (although the phenology of *M brassicae* is currently unknown). Formulation advances, such as microencapsulation or plant‐oil carriers, may localize synergist delivery to pest feeding sites and reduce environmental drift.^67^ Likewise, continued toxicity screening should be expanded to other non‐target arthropods, and efficacy tested under semi‐field or field conditions that include natural enemies. Incorporating such evaluations into IPM programmes will help ensure that pest control measures do not compromise the biological control providers we aim to support.

## 5 CONCLUSION

SYN‐A shows clear promise as a resistance‐breaking tool that could restore pyrethroid efficacy and substantially reduce insecticide usage against CSFB. Although the current study provides evidence of its potential, the conclusions are based on a limited beetle numbers and should be validated through larger‐scale, field‐based experiments. The use of this and other naturally derived synergists is aligned with IPM principles and could allow minimization of toxicant insecticide use; our data suggest it could cut lambda‐cyhalothrin inputs by 80% while maintaining high control levels. Nevertheless, the high toxicity to parasitoids emphasizes that synergists must be used with caution. With careful evaluation and implementation (e.g. precise timing, targeted formulations, complementary preventive tactics) and thorough ecological assessment, SYN‐A and other naturally derived synergists could become a useful tool in sustainable pest management offering a promising avenue for enhancing insecticide efficacy, if its effects on non‐target organisms are evaluated and kept to the minimum.

## CONFLICT OF INTEREST

The authors declare no conflicts of interest. SYN‐A is covered by patent WO2017/005728 held by ApresLabs Ltd.

## Data Availability

The data that support the findings of this study are available from the corresponding author upon reasonable request.
